# Effect of audio instruction on tracking errors using a four‐dimensional image‐guided radiotherapy system

**DOI:** 10.1120/jacmp.v14i5.4488

**Published:** 2013-09-06

**Authors:** Mitsuhiro Nakamura, Akira Sawada, Nobutaka Mukumoto, Kunio Takahashi, Takashi Mizowaki, Masaki Kokubo, Masahiro Hiraoka

**Affiliations:** ^1^ Department of Radiation Oncology and Image‐applied Therapy Graduate School of Medicine Kyoto University Kyoto Japan; ^2^ Department of Radiological Technology Faculty of Medical Science Kyoto College of Medical Science Kyoto Japan; ^3^ Advanced Mechanical Systems Department Mitsubishi Heavy Industries Ltd. Hiroshima Japan; ^4^ Department of Radiation Oncology Kobe City Medical Center General Hospital Hyogo Japan; ^5^ Division of Radiation Oncology Institute of Biomedical Research and Innovation Hyogo Japan

**Keywords:** Vero4DRT, dynamic tumor tracking, tracking accuracy, audio instruction, respiration, phantom study

## Abstract

The Vero4DRT (MHI‐TM2000) is capable of performing X‐ray image‐based tracking (X‐ray Tracking) that directly tracks the target or fiducial markers under continuous kV X‐ray imaging. Previously, we have shown that irregular respiratory patterns increased X‐ray Tracking errors. Thus, we assumed that audio instruction, which generally improves the periodicity of respiration, should reduce tracking errors. The purpose of this study was to assess the effect of audio instruction on X‐ray Tracking errors. Anterior‐posterior abdominal skin‐surface displacements obtained from ten lung cancer patients under free breathing and simple audio instruction were used as an alternative to tumor motion in the superior‐inferior direction. First, a sequential predictive model based on the Levinson‐Durbin algorithm was created to estimate the future three‐dimensional (3D) target position under continuous kV X‐ray imaging while moving a steel ball target of 9.5 mm in diameter. After creating the predictive model, the future 3D target position was sequentially calculated from the current and past 3D target positions based on the predictive model every 70 ms under continuous kV X‐ray imaging. Simultaneously, the system controller of the Vero4DRT calculated the corresponding pan and tilt rotational angles of the gimbaled X‐ray head, which then adjusted its orientation to the target. The calculated and current rotational angles of the gimbaled X‐ray head were recorded every 5 ms. The target position measured by the laser displacement gauge was synchronously recorded every 10 msec. Total tracking system errors $\left(E^{T}\right)$ were compared between free breathing and audio instruction. Audio instruction significantly improved breathing regularity (p < 0.01). The mean ± standard deviation of the 95th percentile of $E^{T}\;(E_{95}^{T})$ was 1.7 ± 0.5 mm (range: 1.1–2.6 mm) under free breathing ($E_{95}^{T,\text{FB}}$) and 1.9 ± 0.5 mm (range: 1.2–2.7 mm) under audio instruction $\left(E_{95}^{T,\text{AI}}\right)$. $E_{95}^{T,\text{AI}}$ was larger than $E_{95}^{T,\text{FB}}$ for five patients; no significant difference was found between $E_{95}^{T,\text{FB}}$ and $E_{T,\text{AI}}^{95}\left(p\;=\;0.21\right)$. Correlation analysis revealed that the rapid respiratory velocity significantly increased $E_{95}^{T}$. Although audio instruction improved breathing regularity, it also increased the respiratory velocity, which did not necessarily reduce tracking errors.

PACS number: 87.55.ne, 87.57.N‐, 87.59.C‐,

## I. INTRODUCTION

Respiratory motion is one of the most important issues to be addressed in radiotherapy.^(^ ^1^ ^,^ ^2^ ^)^ Respiratory motion broadens the dose distribution in the anatomy moving near the beam edges for conventional radiotherapy with uniform radiation intensity^(3)^ and significantly degrades the dosimetric advantage of intensity‐modulated radiotherapy due to the interplay between the motion of a multileaf collimator (MLC) and respiratory motion^(^ ^4^ ^,^ ^5^ ^)^ These impacts can be strongly enhanced, particularly for hypofractionated radiotherapy.

The American Association of Physicists in Medicine Task Group 76 has suggested several approaches to overcome the above shortcomings induced by respiratory motion, such as breath‐holding, respiratory‐gating, and dynamic tumor‐tracking (DTT) techniques.^(6)^ Of these techniques, DTT has recently been of particular interest. DTT detects the tumor location and repositions the MV beam to the target in real time, without a prolonged treatment time or the burden of breath‐holding for patients.

**Figure 1:**
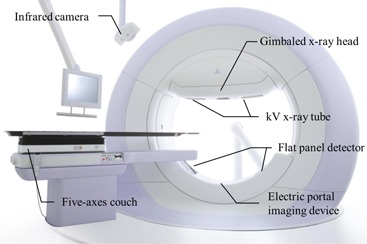
Schematic diagram of the Vero4DRT.

We have developed a four‐dimensional image‐guided radiation therapy system with a gimbaled X‐ray head, the Vero4DRT (MHI‐TM2000) (Mitsubishi Heavy Industries, Ltd., Tokyo, Japan; BrainLAB, Feldkirchen, Germany)^(^ ^7^ ^,^ ^8^ ^)^ (Fig. 1). This system has three special features: 1) an O‐ring‐shaped gantry, 2) a gimbaled X‐ray head, and 3) orthogonal kV X‐ray imaging subsystems. The Vero4DRT can separately rotate the gantry along an O‐shaped guide lane and the O‐ring along its vertical axis, providing noncoplanar three‐dimensional (3D) conformal beam delivery without a treatment couch rotation. The gimbaled X‐ray head, which comprises a compact 6 MV linear accelerator with a C‐band klystron and system‐specific MLC,^(9)^ is mounted on the inside of the O‐ring‐shaped gantry. The gimbaled X‐ray head can rotate along two orthogonal gimbals — pan (horizontal to the O‐ring‐shaped gantry) and tilt (vertical to the O‐ring‐shaped gantry) rotations — up to ± 2.5° with a maximum rotational speed of 9°/sec. By swinging the gimbaled head, the MV beam can be quickly repositioned around the isocenter. Additionally, two orthogonal sets of kV X‐ray tubes and flat panel detectors (FPDs) with a spatial resolution of 0.2 mm at the isocenter level are mounted in the O‐ring‐shaped gantry to simultaneously acquire arbitrary orthogonal fluoroscopic images.^(10)^

The Vero4DRT is capable of performing X‐ray image‐based tracking (X‐ray Tracking) that directly tracks the target or fiducial markers under continuous kV X‐ray imaging. Previously, we verified X‐ray Tracking errors using a 3D movable phantom, which showed that irregular respiratory patterns reduced tracking accuracy.^(11)^ It is generally known that audio instruction improves the regularity of respiratory patterns.^(^ ^12^ ^,^ ^13^ ^)^ Thus, we assumed that audio instruction would improve tracking accuracy. The purpose of the present study was to assess the effect of audio instruction on X‐ray Tracking errors of the Vero4DRT.

## II. MATERIALS AND METHODS

### A. Respiratory motion data

Anterior‐posterior (AP) abdominal skin‐surface displacements obtained from ten lung cancer patients under free breathing and simple audio instruction were used as an alternative to tumor motion in the superior‐inferior (SI) direction. Under audio instruction, all patients were asked to breathe by following the simple audio instruction only, such as “breathe in, breathe out”, at a suitable tempo for each patient.^(13)^ Table 1 shows the peak‐to‐peak motion amplitude (A) and the breathing period (T) of the respiratory pattern for each patient, under free breathing and under audio instruction. The coefficient of variation (CV), defined as the radio of the standard deviation (SD) to the mean, was used to evaluate the regularity of breathing patterns.

**Table 1:** Characteristics of respiratory patterns

	*Free Breathing*						*Audio Instruction*					
	*A*			*T*			*A*			*T*		
*Patient*	*Mean (mm)*	*SD (mm)*	*CV (%)*	*Mean (s)*	*SD (s)*	*CV (%)*	*Mean (mm)*	*SD (mm)*	*CV (%)*	*Mean (s)*	*SD (s)*	*CV (%)*
1	10.9	2.9	26.6	3.9	0.5	12.8	27.0	3.8	14.1	5.5	0.9	16.4
2	8.5	0.6	7.1	3.1	0.2	6.5	18.0	1.8	10.0	3.9	0.1	2.6
3	14.9	2.5	16.8	4.7	0.4	8.5	12.3	0.7	5.7	4.3	0.2	4.7
4	9.3	4.9	52.7	3.7	0.7	18.9	16.8	1.1	6.5	5.1	0.3	5.9
5	7.0	2.5	35.7	3.0	0.5	16.7	15.7	0.8	5.1	5.1	0.3	5.9
6	12.1	6.4	52.9	4.7	1.8	38.3	19.5	1.0	5.1	5.1	0.1	2.0
7	8.4	0.6	7.1	3.5	0.6	17.1	27.6	1.7	6.2	4.9	0.3	6.1
8	5.4	2.3	42.6	3.0	0.7	23.3	16.2	2.2	13.6	4.7	0.3	6.4
9	7.9	1.6	20.3	3.7	0.5	13.5	19.6	1.9	9.7	5.3	0.2	3.8
10	8.6	1.5	17.4	3.5	0.5	14.3	23.8	1.4	5.9	5.3	0.2	3.8

A = peak‐to‐peak motion amplitude; T = breathing period; SD = standard deviation; CV = coefficient of variance.

### B. Experimental system

**Figure 2:**
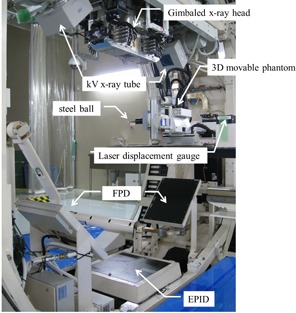
Overview of the experimental system.

Our experimental system comprised a 3D movable phantom with a steel ball target (diameter: 9.5 mm), a laser displacement gauge (positional accuracy: 0.05 mm) used for independent validation of X‐ray Tracking (not part of the Vero4DRT), a kV X‐ray‐imaging subsystem, a gimbaled X‐ray head (stationary accuracy: 0.1 mm), and a system controller for the Vero4DRT (Fig. 2). The positional accuracy of the 3D movable phantom was within ± 0.1 mm.^(14)^ The laser displacement gauge was calibrated by measuring known displacements before following experiments.

First, a sequential predictive model based on the Levinson‐Durbin algorithm^(^ ^15^ ^,^ ^16^ ^)^ was created to estimate the future 3D target position under continuous kV X‐ray imaging by monitoring the moving steel ball target. The prediction accuracy of the predictive model was ± 0.4 mm for a 1D sinusoidal pattern with a peak‐to‐peak motion amplitude of 40 mm and a breathing period of 2 sec in the MHI experiments. While the 3D movable phantom moved with the relevant respiratory pattern, the two‐dimensional (2D) target position was detected on FPD images every 70 msec using the template‐matching technique. The 3D target position was then calculated from a pair of 2D target positions on orthogonal FPD images, using a stereo‐vision technique. Simultaneously, the predictive model was updated automatically from the previous 150 consecutive 3D target positions in real time based on the Levinson‐Durbin algorithm.^(^ ^11^ ^,^ ^15^ ^,^ ^16^ ^)^ The predictive model‐building time was set to 40 sec, and the kV X‐ray imaging parameters were set to 70 kVp, 100 mA, and 5 msec per shot. The time required for image acquisition and image processing was determined to be 66 msec.

After creating the predictive model, the future 3D target position was sequentially predicted from the current 3D target position based on the predictive model every 70 msec under continuous kV X‐ray imaging. The system controller concurrently calculated the corresponding pan and tilt rotational angles of the gimbaled X‐ray head, and then the gimbaled X‐ray head adjusted its orientation to the target every 5 msec. The calculated and current rotational angles of the gimbaled X‐ray head were recorded every 5 msec. Meanwhile, the target position was measured with the laser displacement gauge every 10 msec for independent validation. The laser displacement signals were then sent to the system controller as the true target position for synchronization of the data recordings. Table 2 summarizes the key parameters of the X‐ray Tracking system.

**Table 2:** Key parameters of the X‐ray Tracking

*Model Building*	
kV X‐ray imaging parameters	70 kVp, 100 mA, 5 msec
Predictive model‐building time	40 sec
*Target Detection and Image Processing*	
kV X‐ray imaging parameters	70 kVp, 100 mA, 5 msec
Detection of the 2D target position on FPD images	70 msec
Time to predict the future 3D target position	70 msec
Time required for imaging acquisition and image processing	66 msec
*Mechanical Response Time of the Gimbaled X‐ray Head*	
Frequency of repositioning the gimbaled X‐ray head	5 msec
*Measurement and Data Recording*	
Target position measurement with the laser displacement gauge	10 msec
Recording the rotational angles of the gimbaled X‐ray head	5 msec

2D = two‐dimensional; 3D = three‐dimensional; FPD = flat panel detector.

### C. Data analysis

The predicted target positional errors $\left(E^{P}\;=\;y^{p}\;‐\;y^{m}\right)$, the mechanical response error of the gimbaled X‐ray head $\left(E^{M}\;=\;y^{t}\;‐\;y^{p}\right)$, and the total tracking system errors $\left(E^{T}\;=\;y^{t}\;‐\;y^{m}\right)$ were calculated from the log files. $y^{p},\;y^{m}$, and $y^{t}$ were defined as the target position predicted by the predictive model, measured by the laser displacement gauge, and tracked by the gimbaled MV X‐ray head, respectively. Details of the predictive model and the definitions of $E^{T},\;E^{M}$, and $E^{T}$ have been reported elsewhere.^(11)^

Additionally, the Pearson product‐moment correlation coefficients between the 95th percentile of $E^{T}\;(E_{95}^{T})$ and the characteristics of the respiratory patterns, including the motion amplitude, breathing period, respiratory velocity (v), and respiratory acceleration (a), were analyzed. The correlation coefficient was an indicator of the linear correlation between two variables, which is defined as the covariance of the two variables divided by the product of their SD. The respiratory velocity was calculated from two consecutive target positions, and the respiratory acceleration from two consecutive respiratory velocity values. The two‐sided Wilcoxon test with a 0.05 significance level was performed for statistical analysis.

## III. RESULTS

### A. Effect of audio instruction on respiratory

As seen from the CV of A and T, audio instruction significantly improved breathing regularity (p < 0.01) (Table 1). Audio instruction mostly led to an increase in respiratory velocity (Table 3). The average ratios of the 90th $\left(v_{90}\right)$ percentile of respiratory velocity under audio instruction to those under free breathing were 1.6 (range: 0.8–2.0). Conversely, the average ratios of the 90th $(\alpha_{90}$) percentile of respiratory acceleration under audio instruction to those under free breathing were 1.0 (range: 0.7–1.3) (Table 3). There were also significant differences in respiratory velocity between free breathing and audio instruction (p < 0.01). The strong correlations^(17)^ between the ratio of the mean of the motion amplitude to the mean of the breathing period $\left(Ā/\overset{‐}{T}\right)$ and respiratory velocity were shown under audio instruction (R = 0.95).

**Table 3:** Respiratory velocity and acceleration under free breathing and audio instruction

	*Free Breathing*		*Audio Instruction*		*Ratio*	
*Patient*	$v_{90}\;(\text{mm}/\text{sec})$	$\alpha_{90}\;(\text{mm}/{\text{sec}}^{2})$	$v_{90}\;(\text{mm}/\text{sec})$	$\alpha_{90}\;(\text{mm}/{\text{sec}}^{2})$	$v_{90}^{\text{AI}}/v_{90}^{\text{FB}}$	$\alpha_{90}^{\text{AI}}/v_{90}^{\text{FB}}$
1	11.7	71.4	21.8	94.4	1.9	1.3
2	10.2	76.1	20.5	73.8	2.0	1.0
3	13.2	61.2	11.1	55.1	0.8	0.9
4	13.6	102.0	12.9	73.7	0.9	0.7
5	9.3	72.7	11.8	68.9	1.3	1.0
6	10.9	86.7	13.8	91.8	1.3	1.1
7	10.2	76.5	20.2	84.2	2.0	1.1
8	7.5	81.8	13.5	68.9	1.8	0.8
9	8.4	68.9	15.2	71.4	1.8	1.0
10	9.3	67.0	17.3	76.5	1.9	1.1
mean	10.4	76.4	15.8	75.9	1.6	1.0
SD	2.0	11.6	3.9	11.7	0.5	0.2
max	13.6	102.0	21.8	94.4	2.0	1.3
min	7.5	61.2	11.1	55.1	0.8	0.7

$v_{90}^{\text{AI}}=90\text{th}$ percentile of the respiratory velocity under audio instruction; $v_{90}^{\text{FB}}=90\text{th}$ percentile of the respiratory velocity under free breathing; $\alpha_{90}^{\text{AI}}=90\text{th}$ percentile of the respiratory acceleration under audio instruction; $\alpha_{90}^{\text{FB}}=90\text{th}$ percentile of the respiratory acceleration under free breathing.

### B. Comparison of tracking errors under free breathing and audio instruction

**Figure 3:**
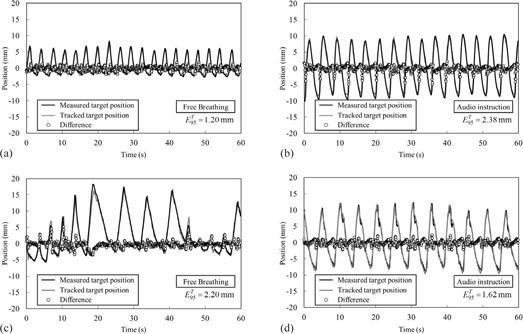
Trajectories of the measured target position (black) and the tracked target position (grey): (a) free breathing for patient 2, (b) audio instruction for patient 2, (c) free breathing for patient 6, and (d) audio instruction for patient 6. The circles indicate the difference between the measured and tracked target positions.

Table 4 summarizes the 95th percentile of absolute $E^{P},\;E_{M}$, and $E^{T}$ for each patient. $E^{P}$ occupied most of the $E^{T}$, whereas $E^{M}$ was negligible. For the entire patient population, the mean ± SD of the $E_{95}^{T}$ was 1.7 ± 0.5 mm (range: 1.1–2.6 mm) under free breathing $\left(E_{95}^{T,\text{FB}}\right)$ and 1.9 ± 0.5 mm (range: 1.2–2.7 mm) under audio instruction $\left(E_{95}^{T,\text{AI}}\right)$. $E_{95}^{T,\text{AI}}$ was larger than $E_{95}^{T,\text{FB}}$ for five patients, and there was no significant difference between $E_{95}^{T,\text{FB}}$ and $E_{95}^{T,\text{AI}}\;(p\;=\;0.21)$. Figure 3 shows trajectories of the measured and tracked target positions for patient 2 (Figs. 3(a) and (b)) and patient 6 (Figs. 3(c) and (d)) under free breathing and audio instruction. The local maximal errors commonly appeared around the peaks positions for irregular respiratory pattern (Fig. 3(c)) and beyond the peak under audio instruction (Figs. 3(b) and (d)).

**Table 4:** $E_{95}^{P}E_{95}^{M}$, and $E_{95}^{T}$ under free breathing and audio instruction

	*Free Breathing*			*Audio Instruction*		
*Patient*	$E_{95}^{P}\;(\text{mm})$	$E_{95}^{M}\;(\text{mm})$	$E_{95}^{T}\;(\text{mm})$	$E_{95}^{P}\;(\text{mm})$	$E_{95}^{M}\;(\text{mm})$	$E_{95}^{T}\;(\text{mm})$
1	1.6	0.1	1.7	2.6	0.3	2.6
2	1.2	0.2	1.2	2.3	0.2	2.4
3	1.7	0.2	1.7	1.4	0.1	1.4
4	2.6	0.2	2.6	1.5	0.2	1.5
5	1.4	0.1	1.4	1.2	0.1	1.2
6	2.2	0.1	2.2	1.7	0.1	1.6
7	1.6	0.1	1.6	2.0	0.2	2.0
8	1.7	0.1	1.7	1.6	0.1	1.6
9	1.1	0.1	1.1	2.6	0.2	2.7
10	1.6	0.2	1.6	2.1	0.2	2.1
mean	1.7	0.1	1.7	1.9	0.2	1.9
SD	0.4	0.0	0.5	0.5	0.1	0.5
max	2.6	0.2	2.6	2.6	0.3	2.7
min	1.1	0.1	1.1	1.2	0.1	1.2

$E_{95}^{P}\;=\;95\;\text{th}$ percentile of target prediction errors; $E_{95}^{M}\;=\;95\;\text{th}$ percentile of mechanical response errors of the gimbaled X‐ray head; $E_{95}^{T}\;=\;95\text{th}$ percentile of total tracking system errors; SD = standard deviation.

### C. Correlations between the characteristics of respiratory patterns and tracking errors

**Figure 4:**
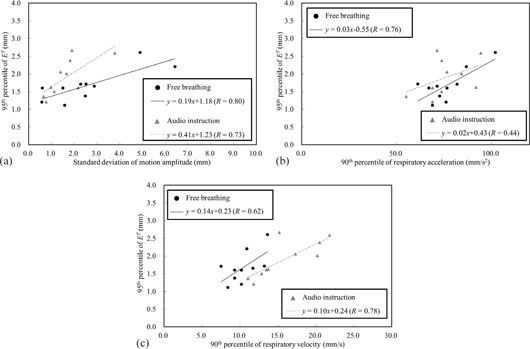
Variations in $E_{95}^{T}$ as a function of (a) ${\text{SD}}_{A}$, (b) $\alpha_{90}$, and (c) $v_{90}$.

Table 5 shows the correlations between $E_{95}^{T}$ and the characteristics of respiratory patterns. It was found that the SD of motion amplitude (${\text{SD}}_{A}$), as well as respiratory velocity and respiratory acceleration, were significantly correlated with $E_{95}^{\text{Tz}}$. In addition, the ${\text{SD}}_{A}$ was highly correlated with $\alpha_{90}$ under free breathing (R = 0.60). Figure 4 shows variations in $E_{95}^{T}$ as a function of ${\text{SD}}_{A},\alpha_{90}$, and $v_{90}$. From the regression lines, $E_{95}^{T}\;<\;2\;\text{mm}$ was estimated from ${\text{SD}}_{A}$ under free breathing $({\text{SD}}_{A}^{\text{FB}})\;<\;4.3\;\text{mm}$ (Fig. 4(a)), $\alpha_{90}$ under audio instruction $(\alpha_{90}^{\text{AI}})\;<\;78.5\;\text{mm}/s^{2}$ (Fig. 4(b)), and $v_{90}$ under audio instruction $(v_{90}^{\text{AI}})\;<\;17.6\;\text{mm}/s$ (Fig. 4(c)), respectively.

**Table 5:** Correlation between the $E_{95}^{T}$ and the characteristics of respiratory patterns

		*Free Breathing*		*Audio Instruction*	
*Parameter*		*R*	*P*	*R*	*P*
*A (mm)*	Mean	0.32	0.36	0.62	0.05
	SD	0.80	< 0.05	0.73	< 0.05
*T (sec)*	Mean	0.43	0.21	0.13	0.71
	SD	0.56	0.09	0.37	0.30
$v_{90}\;(\text{mm}/\text{sec})$		0.62	0.06	0.78	< 0.05
$\alpha_{90}\;(\text{mm}/{\text{sec}}^{2})$		0.76	< 0.05	0.44	0.21

$E_{95}^{T}\;=\;95\text{th}$ percentile of total tracking system errors; A = peak‐to‐peak motion amplitude; T = breathing period; SD = standard deviation; $v_{90}\;=\;90\text{th}$ percentile of the respiratory velocity; $\alpha_{90}\;=\;90\text{th}$ percentile of respiratory acceleration.

**Figure 5:**
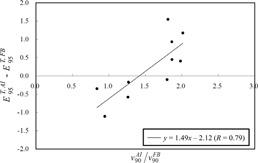
Relationship between the difference in the $E_{95}^{T}$ and the ratio of $v_{90}$. The horizontal axis shows the ratio of $v_{90}$ under audio instruction to that under free breathing. The vertical axis shows the difference of $E_{95}^{T}$ under free breathing from that under audio instruction.

There was a strong correlation between the difference in the $E_{95}^{T}$ and the $v_{90}$ ratio (Fig. 5). The horizontal axis shows the ratio of $v_{90}$ under audio instruction to that under free breathing, and the vertical axis the subtraction of $E_{95}^{T}$ under free breathing from that under audio instruction. Negative values on the vertical axis indicate that the $E_{95}^{T}$ under audio instruction was smaller than those under free breathing. Figure 5 shows that the tracking accuracy was mostly reduced, with a $v_{90}$ ratio of > 1.4, even for regular respiratory patterns under audio instruction.

## IV. DISCUSSION

We assumed that audio instruction would improve the tracking accuracy; however, our hypothesis that tracking errors were reduced with audio instructions did not hold for all cases. Audio instruction mostly led to an increase in respiratory velocity, which could be a factor in preventing reduction in X‐ray Tracking errors.

Shirato et al.^(18)^ previously showed that the average maximum speed of the implanted fiducial markers was 21.1 ± 18.9 mm/sec using a respiratory‐gating radiotherapy system, and suggested that the high respiratory velocity may make realization of DTT irradiation difficult. Wijesooriya et al.^(19)^ also indicated that the respiratory velocity was one of the crucial factors for inducing beam hold for dynamic MLC‐based DTT irradiation. In the current study, despite the maximum $v_{90}$ of 21.8 mm/sec, $E_{95}^{M}$ was up to 0.3 mm under audio instruction (Table 4), indicating that the mechanical response of the gimbaled X‐ray head was favorable even for faster respiratory motion.

It was found that $E_{95}^{T}$ had a strong correlation with ${\text{SD}}_{A}$ under free breathing (Table 5 and Fig. 4(a)). This result can be explained from the property of the predictive model. As the target position nears its peak, the predictive model needs to calculate the next target position beyond the peak, based on the previous target positions. Especially for irregular respiratory patterns, it may be difficult to predict the next position around the peak with high accuracy, which results in pronounced errors around the peak, as shown in Fig. 3(c). Additionally, $\alpha_{90}$ was a factor to estimate the tracking error under free breathing (Fig. 4(b)). In general, $\alpha_{90}$ was derived from sudden changes in respiratory patterns, such as deep breathing, hiccup, and cough. These unexpected changes led to an increase in ${\text{SD}}_{A}$ for irregular respiratory patterns (R = 0.60 ). Although $E_{95}^{T,\text{FB}}$ was 2.61 mm, despite irregular respiratory patterns, a higher tracking accuracy is expected for small ${\text{SD}}_{A}^{\text{FB}}$, as shown in Fig. 4(a). Figures 3(c) and (d) illustrate this point.

Audio instruction decreased the CV of respiratory amplitude and breathing period, which led to improvement of the breathing regularity (Table 1); however, it also increased the respiratory amplitude, which led to increase of the respiratory velocity (Table 3). Correlation analysis revealed that $v_{90}$ was a predictor of $E_{95}^{T}$ under audio instruction (Table 5 and Fig. 4(c)). For respiratory motions with a high velocity, the predictive model cannot anticipate the future target position accurately. The tracking errors were observed around the peak positions of the target due to the difficulty of prediction (Figs. 3(b) and (d)). In general, respiratory velocity is proportional to A/T when the regular respiratory pattern is expressed as *A* sin(2π /*T*‐ φ). We observed that $v_{90}^{\text{AI}}$ was highly correlated with $Ā/\overset{‐}{T}\;(R\;=\;0.95)$. In general, the breathing period for patients ranged from 3 to 6 sec,^(20)^ which was comparable to our study with and without audio instruction. Therefore, controlling the motion amplitude is effective for the reduction of $E_{95}^{T}$. Additionally, Figs. 4(c) and 5 provided an upper limit of $v_{90}$ to achieve a higher tracking accuracy for regular respiratory patterns. From these findings, respiratory instruction techniques that control the increasing motion amplitude while maintaining high regularity of the respiratory patterns, such as audio‐visual coaching,^(21)^ will be useful for reducing $E_{95}^{T}$ for X‐ray Tracking.

Two limitations of our study warrant mention. The first is moving direction. Our study was limited to the SI direction only because we measured the target position only in the SI direction with the laser displacement gauge due to a structural issue. The SI direction is generally known to be the predominant direction of breathing motion.^(6)^ Even if the target moves three‐dimensionally, the tracking accuracies would be high, according to the results of our previous study.^(11)^ The second limitation concerns the phantom study. Our study was limited to the phantom with a steel‐ball target. The projected lung tumor shape and appearance vary more or less during breathing for real patients. However, simulated target motion used in our study was acquired from real patients. If a tumor itself or internal fiducials are clearly identified, similar results would be obtained even for real patients.

## V. CONCLUSIONS

We investigated X‐ray Tracking errors of the Vero4DRT under free breathing and audio instruction while moving the target in the SI direction. Audio instruction improved breathing regularity; however, it also increased the respiratory velocity. Additionally, there was a strong correlation between $v_{90}$ and $E_{95}^{T}$ under audio instruction. Therefore, respiratory instruction techniques that control the increasing motion amplitude, while retaining high regularity in the respiratory patterns, can achieve high‐tracking accuracy.

## ACKNOWLEDGMENTS

This research was funded by the Japan Society for the Promotion of Science (JSPS) through the “Funding Program for World‐Leading Innovative R&D on Science and Technology (FIRST Program)”, invited by the Council for Science and Technology Policy (CSTP). We would like to express our appreciation to the entire technical staff at Mitsubishi Heavy Industries, Japan, for data acquisition.
